# A multi-centre evaluation of oral cancer in Southern and Western Nigeria: an African oral pathology research consortium initiative

**DOI:** 10.11604/pamj.2017.28.64.13089

**Published:** 2017-09-22

**Authors:** Olufemi Gbenga Omitola, Olujide Oladele Soyele, Opeyemi Sigbeku, Dickson Okoh, Abdulwarith Olaitan Akinshipo, Azeez Butali, Henry Ademola Adeola

**Affiliations:** 1 Department of Oral Pathology and Biology, University of Port Harcourt, Port Harcourt, Nigeria; 2 Department of Oral Maxillo-Facial Surgery and Oral Pathology, Obafemi Awolowo University, Ile-Ife, Nigeria; 3 Department of Oral Pathology, College of Medicine, University of Ibadan, Ibadan, Nigeria; 4 Department of Oral Pathology, School of Dentistry, University of Calabar, Nigeria; 5 Department of Oral and Maxillofacial Pathology & Biology, College of Medicine, University of Lagos, Lagos, Nigeria; 6 Department of Oral Pathology, Radiology and Medicine, University of Iowa, Iowa City, IA, USA; 7 Department of Oral and Maxillofacial Pathology, Faculty of Dentistry, University of the Western Cape and Tygerberg Hospital, Cape Town, South Africa; 8 Division of Dermatology, Department of Medicine, Faculty of Health Sciences and Groote Schuur Hospital, University of Cape Town, Cape Town, South Africa

**Keywords:** Oral cancer, multi-centre, Nigeria, AOPRC, oral pathology

## Abstract

**Introduction:**

Oral cancer is a leading cause of cancer deaths among African populations. Lack of standard cancer registries and under-reporting has inaccurately depicted its magnitude in Nigeria. Development of multi-centre collaborative oral pathology networks such as the African Oral Pathology Research Consortium (AOPRC) facilitates skill and expertise exchange and fosters a robust and systematic investigation of oral diseases across Africa.

**Methods:**

In this descriptive cross-sectional study, we have leveraged the auspices of the AOPRC to examine the burden of oral cancer in Nigeria, using a multi-centre approach. Data from 4 major tertiary health institutions in Western and Southern Nigeria was generated using a standardized data extraction format and analysed using the SPSS data analysis software (version 20.0; SPSS Inc. Chicago, IL).

**Results:**

Of the 162 cases examined across the 4 centres, we observed that oral squamous cell carcinomas (OSCC) occurred mostly in the 6^th^ and 7^th^ decades of life and maxillary were more frequent than mandibular OSCC lesions. Regional variations were observed both for location, age group and gender distribution. Significant regional differences was found between poorly, moderately and well differentiated OSCC (p value = 0.0071).

**Conclusion:**

A multi-centre collaborative oral pathology research approach is an effective way to achieve better insight into the patterns and distribution of various oral diseases in men of African descent. The wider outlook for AOPRC is to employ similar approaches to drive intensive oral pathology research targeted at addressing the current morbidity and mortality of various oral diseases across Africa.

## Introduction

Oral cancer is the sixth commonest cancer globally and the most common head and neck cancer [1]. Oral squamous cell carcinoma (OSCC) are cancers originating from the squamous epithelium in the oral cavity, accounting for more than 90% of all tumors in the head and neck region [2–4]. According to the World Health Organization (WHO)'s International Classification of Diseases (ICD-10), “Oral cancer” (C00, C02-C06) may be defined as any malignant neoplasm occurring on the lips (both vermillion border and oral aspect) and within the oral cavity (which includes the anterior 2/3 of the tongue, buccal and labial mucosa, gingiva, hard palate, retromolar pad and floor of the mouth) [5]. There is a marked variation with regard to the incidence of OSCC between different countries, geographic locations and ethnic/racial groups [6]. This may be attributed to exposure to different environmental factors and to ethnic-specific high-risk habits [6]. The global average incidence from the WHO/IARC global cancer statistics database (GLOBOCAN 2012) shows “oral cancer” to be the sixteenth commonest cancers type, accounting for 300,200 new cases, which consisted of 198,900 and 101,300 new cases among males and females, respectively [7]. According to this database, oral cancer related deaths has been estimated to be about 145,353 cases with about 97,900 cases for males and 47,400 cases for females in 2012; making it the twelfth and sixteenth most common cause of cancer mortality amongst males and females, respectively. In Nigeria, oral cancer has been estimated to account for about 1146 new cases, with an estimated mortality of 764 cases annually in 2012 [7].

The aetiology of oral cancer although unknown, has been historically associated with the use of tobacco products, alcohol consumption, infections with Human Papilloma virus (HPV) and environmental carcinogens. In developed regions 75% of oral cancer cases may be linked to the use of tobacco and alcohol consumption by patients [8]. However, in developing countries, other risk factor such as use of betel quid, consumption of nitrosamine rich foods (such as salted fish), infections, nutritional deficiencies and exposure to environmental carcinogens may account for the differences in epidemiology of oral cancers in different regions [9, 10]. The epidemiology of Lip, and oral cavity cancers also vary across regions with India, USA and China accounting for the highest prevalence and incidence rates. In India, incidence of oral cancer has been reported to be about 11% of all cancers in 2012, while prevalence has been reported to be about 12%, making oral cancer the commonest cancers in males and the seventh in females in India [11]. However, due to underreporting, it is unclear if the burden of oral cancer is actually low in Nigeria or the low figures is as a result of poorly documented cases and lack of well-funded, state-of-the-art, population-based cancer registries across the country [12]. Most cancer registries in Nigeria are hospital-based and lack adequate coverage and reliability. Thus, there is a critical need to have a better understanding of the real distribution of oral cancer as they constitute a serious public health concern globally [11]. The African Oral Pathology Research Consortium (AOPRC) was inaugurated during the 1^st^ regional congress of the International Association of Oral Pathologists (IAOP), which took place in Lagos, Nigeria in September 2015. The objectives for creating AOPRC includes: advancing multi-centre collaborative oral pathology research; exchange of skills and expertise; and strengthening ties between oral pathologists across Africa and further afield, *inter alia*. Accordingly, this study aims to describe and compare the burden of oral cancers using a collaborative, multi-centre, team science approach.

## Methods

**AOPRC participants**: The African Oral Pathology Research Consortium (AOPRC) was inaugurated during the 1^st^regional congress of the International Association of Oral Pathologists (IAOP), which took place in Lagos, Nigeria in September 2015. The objectives for creating AOPRC includes: advancing multi-centre collaborative oral pathology research; exchange of skills and expertise; and strengthening ties between oral pathologists across Africa and further afield, *inter alia*. The AOPRC currently have members based at the University of Benin, Nigeria; University of Ibadan, Nigeria; University of Port Harcourt, Nigeria, University of Calabar, Nigeria; University of Lagos, Nigeria; Obafemi Awolowo University, Ile-Ife, Nigeria; University of Cape Town, South Africa; University of the Western Cape at Tygerberg Hospital, South Africa; and University of Iowa, USA. We have leveraged the auspices of the AOPRC to examine the burden of oral cancer in Nigeria, using a multi-centre approach.

**Data source**: De-identified secondary archived data from four major tertiary health institutions in western and Southern Nigeria were used in this study. Records of all OSCC patients in the University of Port Harcourt, University of Ibadan, Obafemi Awolowo University (Ile-Ife) and University of Benin were extracted from 1990-2016 (16 years), using a standardized data extraction format across all four centres. The records contains demographic data as well as histopathological data such as: age distribution, site, gender distribution, location and histological subtype, *inter alia*.

**Case selection and exclusion criteria**: Cases without adequate clinical and histopathological information were excluded from the study. A total of 162 qualified OSCC cases were selected for this study from the four participating centres. Cases were classified according to the 10^th^ revision of the International Classification of Diseases (ICD-10) regional classification. Lesions of the hypopharynx, nasopharynx, larynx, other pharynx and hematological system were excluded. Carcinoma-in-situ were excluded as well. Selected cases were categorized by histological types, site of primary tumour, age and gender distribution.

**Data analysis**: Data from the selected 162 cases were collated and processed using the SPSS data analysis software (version 20.0; SPSS Inc. Chicago, IL). Categorical variables were analyzed as frequencies and percentages, while quantitative variables were summarized as means and standard deviation.

## Results

**Age distribution**: The mean age of all patients from all the study centres was 56.2 ± 16.4 years. The highest mean age was found in Port Harcourt having a mean age of 65.4 ± 11.9 years, with an age range between 49 to 88 years. While the lowest mean age was observed in Ibadan having a mean age of 54.4 ± 10.5, with age range between 45 to 75 years (Table 1).

**Table 1:** Age distribution of patients with OSCC in the different centers

Summary	Benin	Ibadan	Ife	Port Harcourt	ANOVA
Mean ±SD	55.9±17.3	54.4±10.5	57.2±12.5	65.4±11.9	0.1235
Min	11	45	29	49	
Max	94	75	74	88	
Number of Patients	77	20	46	19	

**Figure 1:**
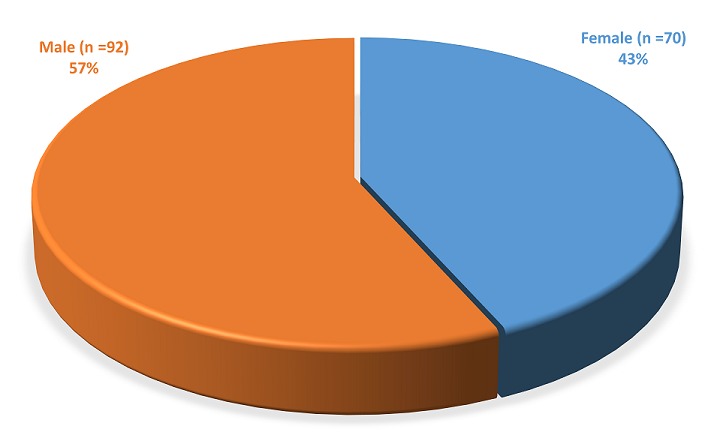
Gender distribution of OSCC among patients: there were more male than female cases

**Gender distribution**: In this study, there were 92 males (57%) and 70 females (43%) with a male to female ratio of 1.3:1 (Figure 1). The male to female ratio were 1.5:1 in Benin, 1:1 in Ibadan, 1:0.9 in Ife and 1.7:1 in Port Harcourt, however the sex distribution of patients with SCC was not significant (p = 0.5023) (Table 2).

**Table 2:** Gender distribution of patients with OSCC

Sex	Benin (n, %)	Ibadan (n, %)	Ife (n, %)	Port Harcourt (n, %)	Chi-Square (*p-*value)
Male	46 (59.7)	10 (50.0)	22 (47.8)	12 (63.2)	2.354 (0.5023)
Female	31 (40.3)	10 (50.0)	24 (52.2)	7 (36.8)	
**Total**	77 (100.0)	20 (100.0)	46(100.0)	19 (100.0)	

**Figure 2:**
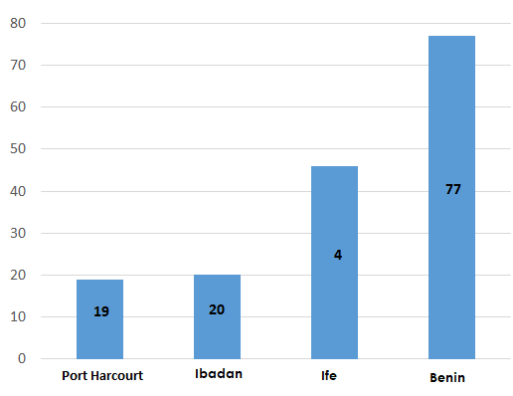
Frequency of OSCC by location; the highest frequency of occurrence of SCC was observed in Benin (n = 77, 47.5%) which was followed by Ife (n = 46, 28.4%); the lowest frequency was seen in Port Harcourt

**Regional distribution**: The highest frequency of occurrence of SCC was observed in Benin (n = 77, 47.5%) which was followed by Ife (n = 46, 28. 4%). The lowest frequency was seen in Port Harcourt (n = 19, 11.7%) (Figure 2).

**OSCC distribution according to age group and location**: The association of SCC with the age groups and location was found to be significant (p value = 0.0293). No occurrence of SCC was observed in the first three decade of life in Ibadan and Port Harcourt. However, in Benin, SCC was seen in patients from the first through the seventh decade of life. In Ife, SCC was observed from the fourth decade, while Port Harcourt and Ibadan found SCC in Patients from the 5^th^ decade of life. Overall, SCC occurred mostly in the 7^th^ decade (n = 69, 45.4%) with the highest frequency in Benin (n = 34, 42.5%) followed by Ife (n = 23, 50.0%). The least occurrence is found in the first, second decade (n = 4, 2.6%) and the third decade (n = 5, 3.3%) with all the 9 cases reported in Benin (Table 3).

**Table 3:** OSCC distribution according to age group and location

Age Group (years)	Benin	Ibadan	Ife	Port Harcourt	Total	Chi-Square (p-value)
<18	4 (5.0)	0 (0.0)	0 (0.0)	0 (0.0)	4 (2.6)	26.93 (0.0293)
18-28	5 (6.3)	0 (0.0)	0 (0.0)	0 (0.0)	5 (3.3)	
29-39	6 (7.5)	0 (0.0)	8 (17.4)	0 (0.0)	14 (9.2)	
40-50	13 (16.3)	4 (57.1)	4 (8.7)	2 (10.5)	23 (15.1)	
50-60	18 (22.5)	2 (28.6)	11 (23.9)	6 (31.6)	37 (24.3)	
>60	34 (42.5)	1 (14.3)	23 (50.0)	11 (57.9)	69 (45.4)	

**Regional histopathological subtype distribution of OSCC**: The histopathologic sub-types seen in all the centres were the well differentiated, moderately differentiated and the poorly differentiated SCC. Overall, the well differentiated SCC (n = 89, 54.9%) was the most common subtype seen while the poorly differentiated (n = 27, 16.7%) was the least common subtype found. Benin (n = 52, 67.5%) and Ife (n = 22, 47.8%) found a high frequency of the well differentiated SCC while Port Harcourt found a slightly higher occurrence of the well differentiated SCC (n = 9, 47.3%) than the moderately differentiated. Ibadan found a slightly higher occurrence of the moderately differentiated SCC (n = 7, 35.0%) than the well differentiated SCC. The distribution of histopathological subtypes of SCC from the different centres were found to be significant (p = 0.0071) (Table 4).

**Table 4:** Distribution of histological subtypes of OSCC

Type	Benin (n, %)	Ibadan (n, %)	Ife (n, %)	Port Harcourt (n, %)	Total (n, %)	Chi-Square (*p-*value)
Poor	6 (7.8)	7 (35.0)	12 (26.1)	2 (10.5)	27(16.7 )	17.69 (0.0071)*
Moderate	19 (24.7)	7 (35.0)	12 (26.1)	8 (42.1)	46(28.4)	
Well-differentiated	52 (67.5)	6 (30.0)	22 (47.8)	9 (47.3)	89(54.9)	
**Total**	77(100.0)	20(100.0)	46(100.0)	19(100.0)	162(100)	

**Site distribution of OSCC**: The maxilla (n = 33, 20.4%) and palate (n = 33, 20.4%) were the most common site. Among the least common sites were the upper lip (n = 2, 1.3%) and parotid (n = 3, 1.9%) (Table 5).

**Table 5:** Distribution of OSCC by site

Site	Frequency (n)	Percent (%)
Parotid	3	1.9
Antrum	7	4.3
Face	5	3.0
Upper Lip	2	1.3
Lower Lip	5	3.0
Floor of Mouth	17	10.5
Cheek	13	8.0
Nasopharynx	10	6.2
Tongue	11	6.8
Mandible	23	14.2
Maxilla	33	20.4
Palate	33	20.4
**Total**	162	100.0

## Discussion

It has been established in the scientific literature that OSCC is more prevalent in developing than developed countries [6, 13]. For instance, In Israel, oral SCC is more prevalent among Ashkenazi Jews than Sephardic-Jews for reason attributed to the differences in geographic origins [14]. Similarly in England, it is more prevalent among Indian people born in the Indian subcontinent and migrated to England than among Indians born in England [15]. It has further been documented in US, that the average 5-year survival rate for men of African descent with OSCC is lower than those of their Caucasian counterparts [6, 16]; and OSCC is usually at a significantly more advanced stage in blacks than in white people at the time of diagnosis [16]. The possible suggestions accrued to these geographical, ethnic and racial differences include; that the lesion is pathobiologically more aggressive in blacks than in whites; and that blacks delay longer before seeking medical advice than do whites for cultural, educational and socioeconomic reasons. Although factors such as socioeconomic status, educational level, cultural influences and limited access to healthcare services may not impact directly on the development of OSCC, however they possess an indirect influence in the higher morbidity and mortality from OSCC [6, 16]. Similar trends were observed in our study, as our result showed that out of the 162 OSCC cases examined across the four centres, the highest incidence was found in Benin which is in the South-south geographical zone of the country, accounting for 47.53% (77). Almost equal number of cases were observed from Ibadan and Port-Harcourt centres viz: 12.35% and 11.73%, respectively. Whereas, the incidence from Ife centre was 28.40%. The observed differences between the four centres could possibly be attributable to disparities in heavy alcohol and tobacco product consumption, as well as low levels of education about the risk factors across the South-South and South-Western geographic zones of Nigeria.

As with many other types of cancer, OSCC most commonly occurs in the middle aged and elderly population [17, 18]. This is in agreement with our findings as majority (69 cases, 42.59%) of the cases were seen within the age range of 60 years and above. This is followed by 37 cases (22.84%) observed between 50-60 years of age. Oral squamous cell carcinoma has long been considered to be a tumor of the elderly and has been seen only sporadically before the third decade of life. In our study, four cases (2.47%) were seen within the age bracket of less than 18 years. Male population have traditionally been known to have a higher incidence in OSCC, typically circa 2:1 compared to women [19, 20]. This trend has however evened out, probably due to increased alcohol consumption and tobacco use among the female population [18]. This development agrees with our findings of male to female ratio of 1.3:1. In addition a male to female ratio of 1.4: 1 has also been reported among Iranian populations [21]. In many studies (including those conducted in Nigeria), OSCC affected more males than females [18, 22–25]. One exception is a study carried out in Ilorin (North Central Nigeria), where females were affected more than males [26]. We also observed in our study that the out of the 46 cases from Ife, 22 of them are females gender with a very close male to female ratio of 1:0.9. We observed that oral squamous cell carcinomas (OSCC) occurred mostly in the 6^th^ and 7^th^ decades of life and maxillary were more frequent than mandibular OSCC lesions. There were more male (n = 92) than female (n = 70) OSCC cases; and regional variations were observed both for location, age group and gender distribution. Our observation is consistent with what others have reported in the literature [27, 28]. For instance Dourmishev et al (1997) reported 2.3:1 male to female ratio and highest frequency of OSCC in patients between 61-70 years [27].

Nyi et al, (2015) reported a much higher ratio of 2.6:1 male to female ratio with the highest frequency of OSCC in patients over 50 years of age [28]. The similarities in trends regardless of population suggest that the underlying molecular signatures and environmental factors that may be increasing the risks are likely the same. Therefore a cohort of an African population with OSCC using genetic and genomic studies to identify risk loci will provide insights into founder mutations since Africa is the ancestral population. The increase in frequency of OSCC with age is likely due to increase in DNA damage with age [29, 30]. This study showed that the most common histological subtype was the well differentiated, followed by the moderately differentiated in all the centres, except Ibadan where well differentiated subtype was the least common. This observation is in agreement with the finding of other researchers who have reported preponderance of well differentiated SCC in their studies [31, 32]. However, Effiom et al (2008) in a study from two centres in Lagos, Nigeria observed that the most predominant subtype was the poorly differentiated and the moderately differentiated type was the least [25]. They also noted that an earlier study from one of the centres reported that that well differentiated subtype was the commonest. The reason for the intra and inter centres variations reported by various researchers is not clear and may require further study. Contrary to several other reports, the sites that were commonly affected in this study were the palate and maxilla followed by the mandible. The mandible and the tongue have been reported as the most favoured site for oral squamous cell carcinoma [25, 31–33], because carcinogens that dissolved in saliva readily settle down to these sites due to gravity. Palate is not a usual site for this lesion, in fact, in the study by Udeabor et al (2014), palate was a distant third most favoured site after mandible and maxilla [33]. The pooling of data from different centres and the inclusion of some additonal oro-facial sites which are usually excluded in other studies may have influence the pattern seen in the present study. Thus, there is need for more elaborate study involving many centres in order to get the true picture of this lesion in our environment.

## Conclusion

The true burden of OSCC as a public health problem among men of African descent cannot be ascertained by merely evaluating public databases. This is particularly true in Nigeria where most data is generated from defunct hospital-based registries rather than population-based repositories. Changing lifestyle patterns due to emergence of many economies in Africa must be factored into the true picture of OSCC burden in Africa. In addition, the aging population has also increased due to better health education and healthcare programs. However, the journey is still long without the ability to leverage partnership with colleagues across the globe, both for diagnostic and research services. A well-coordinated and vibrant cancer control program as it exists in many developed economies should be adopted in Nigeria and many other African countries, to improve prompt diagnosis and management of OSCC. The trends in OSCC identified in this study demonstrated that further multi-centre collaborative research is required to gain better insight into the epidemiology of the disease.

### What is known about this topic

- Oral cancer is a leading cause of cancer deaths in Africa;
- The real burden of oral cancer is poorly captured in Nigeria due to under-reporting and lack of population based registries;
- Collaborative team science in needed among oral pathologist to elucidate the burden of oral cancer in Africa, and advance better diagnostic and treatment modalities.

### What this study adds

- This study presents a multi-centre approach to oral cancer epidemiology in Nigeria;
- We have examine regional variations in oral cancer distribution across the four centres evaluated;
- The multicentre approach to oral cancer study presented herein by AOPRC, would serve as a primer for further collaborative oral pathology research across Africa and globally.

## Competing interests

The authors declare no conflict of interest.
